# Effect of dietary protein content shift on aging in elderly rats by comprehensive quantitative score and metabolomics analysis

**DOI:** 10.3389/fnut.2022.1051964

**Published:** 2022-11-03

**Authors:** Wenxuan Zheng, Ruiding Li, Yang Zhou, Fengcui Shi, Yao Song, Yanting Liao, Fan Zhou, Xiaohua Zheng, Jingwen Lv, Quanyang Li

**Affiliations:** ^1^School of Light Industry and Food Engineering, Guangxi University, Nanning, China; ^2^Wangdingdi Hospital, Tianjin, China

**Keywords:** comprehensive quantitative score, metabolomics, aging, dietary patterns, nuclear magnetic resonance

## Abstract

In the protein nutrition strategy of middle-aged and elderly people, some believe that low protein is good for health, while others believe high protein is good for health. Facing the contradictory situation, the following hypothesis is proposed. There is a process of change from lower to higher ratio of protein nutritional requirements that are good for health in the human body after about 50 years of age, and the age at which the switch occurs is around 65 years of age. Hence, in this study, 50, 25-month-old male rats were randomly divided into five groups: Control (basal diet), LP (low-protein diet with a 30% decrease in protein content compared to the basal diet), HP (high-protein diet with a 30% increase in protein content compared to the basal diet), Model 1 (switched from LP to HP feed at week 4), and Model 2 (switched from LP to HP feed at week 7). After a total of 10 weeks intervention, the liver and serum samples were examined for aging-related indicators, and a newly comprehensive quantitative score was generated using principal component analysis (PCA). The effects of the five protein nutritional modalities were quantified in descending order: Model 1 > HP > LP > Control > Model 2. Furthermore, the differential metabolites in serum and feces were determined by orthogonal partial least squares discriminant analysis, and 15 differential metabolites, significantly associated with protein intake, were identified by Spearman’s correlation analysis (*p* < 0.05). Among the fecal metabolites, 10 were positively correlated and 3 were negatively correlated. In the serum, tyrosine and lactate levels were positively correlated, and acetate levels were negatively correlated. MetaboAnalyst analysis identified that the metabolic pathways influenced by protein intake were mainly related to amino acid and carbohydrate metabolism. The results of metabolomic analysis elucidate the mechanisms underlying the preceding effects to some degree. These efforts not only contribute to a unified protein nutrition strategy but also positively impact the building of a wiser approach to protein nutrition, thereby helping middle-aged and older populations achieve healthy aging.

## Introduction

The global prevalence of obesity has increased markedly, almost tripling in the past few decades, and it has become increasingly common in the elderly (1). Unhealthy lifestyles, especially poor nutritional intake, are to blame. Nutritional imbalances, especially excess food intake, are associated with many severe aging-related diseases, such as diabetes, cardiovascular disease, Alzheimer’s disease, and cancer (2–4). Although dietary restriction may improve obesity in older adults, prolonged dietary restriction is undoubtedly unrealistic because of low adherence. Recent studies have shown that dietary restriction delays aging because of protein restriction rather than total dietary calorie restriction (5–7). Malondialdehyde (MDA), total antioxidant capacity (T-AOC), and interleukin-6 (IL-6) are typical biomarkers that reflect the body’s aging status (8). Several randomized clinical trials and animal studies have found that low-protein (LP) diets are well suited to improve the body’s metabolic health by increasing T-AOC levels, decreasing MDA levels, and lowering IL-6 concentrations, thus demonstrating the benefits of LP diets in combating oxidative stress and improving inflammation levels (9–11). In addition, protein restriction can lower serum insulin growth factor (IGF-1) and enhance insulin sensitivity. Both animal and human studies have shown that normal glucose metabolism, lower insulin levels, and higher insulin sensitivity can constitute markers of healthy aging and longevity (12). Insulin resistance can promote oxidative stress and inflammation through multiple pathways (13), cause cognitive dysfunction (14), accelerate cardiovascular aging (12), and increase the risk of frailty in older populations (15). Protein restriction can alter epigenetic modifications, and such alterations may have long-term effects on gene expression and organ function (16). In terms of epigenetic mechanisms, protein restriction can improve health by affecting transcription and translation as well as gene expression profile (17). For example, protein restriction can inhibit tumor growth through epigenetic modification and inhibition of the IGF/Akt/mTOR pathway (18), and can mediate effects on stem cell function through epigenetic changes (19). Thus, protein restriction can also influence lifespan by affecting epigenetic programs. Various dietary patterns that favor longevity have distinct features of LP diets. In the Okinawan dietary pattern, the proportion of carbohydrate energy supply is as high as 85%, whereas protein accounts for only 9% (20). Low protein intake is also a characteristic of the dietary longevity pattern of Guangxi, China, which is being studied by our team for many years (21).

However, contradictory phenomena have also been observed. Robert et al. reported that protein malnutrition in the elderly causes sarcopenia (22). Arthur et al. found that protein deficiency in the elderly is associated with sarcopenic obesity and cardiovascular diseases (23). Green et al. found that protein restriction promotes metabolic health in mice, but the specific benefits depend on sex, strain, restriction level, and age (24). These results raise questions about whether the elderly should also be on an LP diet. A growing body of evidence suggests that starting or continuing an LP diet later in life may be flawed for some adults over 65 years of age. Morgan et al. studied the relationship between protein intake and mortality from related diseases through a questionnaire in the United States (25). They suggested that “low protein intake in middle-aged people and high protein intake in older people may be beneficial for health and longevity.” In the face of conflicting research results, years of exploration by a combined team led to the following hypothesis: the nutritional protein requirements are not constant after the age of 50, but there is a smooth change from the need for a lower to higher content rates. The switch occurs around the age of 65 years, and the drastic change is detrimental to health.

Some studies have demonstrated that supplementation with a high-protein (HP) diet in protein-malnourished older adults can increase muscle mass (26, 27). However, a short-term switch from an LP to an HP diet may negatively affect older adults with relatively poor self-regulation. Long-term epidemiological studies have shown that HP intake is associated with an increased risk of cardiovascular diseases, diabetes, and mortality (28, 29). The changes in metabolic function, the risk of kidney disease (30), and the risk of age-related diseases such as cancer, associated with abrupt dietary shifts indicate that the dietary patterns of older adults need to be studied more intensively and carefully (31). It is essential to understand the reasons behind the “harmful” and “beneficial” results of recommended LP and HP diets in different age groups. What are the corresponding changes in the body metabolism? Does the rapid transition from an LP to HP diet cause metabolic disturbances in older adults? Research on these questions has rarely been reported. Therefore, it is necessary to explore the physical changes in middle-aged and elderly populations when changing from an LP to HP diet.

Changes and trajectories in metabolic profiles can provide direct and indirect information to explore alterations in metabolism during dietary interventions (32). Therefore, it is essential to use metabolomic techniques to explore the metabolic impact of changes in protein intake. As a downstream application of systems biology, metabolomics can accurately capture changes in the state of the organism and identify possible signals or biomarkers and has become an essential tool for understanding the mechanisms of organismal health. A growing number of studies have shown that metabolites play crucial roles in physiological and pathological aging. Aging and dietary interventions cause changes in metabolites, and alterations in metabolites and metabolic pathways can help to elucidate the mechanisms of diseases or treatments (33). In addition, changes in multiple metabolites have been associated with oxidative stress and inflammation. Several biomarkers such as polyunsaturated fatty acids, glucose, ornithine, arginine, and lactate are associated with dietary habits, apoptosis, mitochondrial dysfunction, inflammation, lipid metabolism, autophagy, and oxidative stress resistance (34, 35), and thus can serve as biomarkers of aging to some extent to reflect the aging status of individuals. Therefore, analyzing metabolomic profiles of feces and serum can provide comprehensive access to overall metabolic information and a strong foundation to explore the effect of protein intake shifts on aging.

Based on this, we aimed to explain the action pattern of protein nutrition by mimicking the human aging process in rats. Based on the intake of isocaloric diets, the diet of rats was changed from LP to HP nutrition in different groups and at different time points. By characterizing aging-related indicators and analyzing non-targeted fecal and serum metabolomic profiles, we verified the accuracy of the above mentioned hypothesis, explored the physical changes in middle-aged and elderly populations when changing from an LP to HP diet, and provide new ideas and references for developing healthy dietary patterns in middle-aged and elderly populations.

## Materials and methods

### Animals and experimental design

**FIGURE 1:**
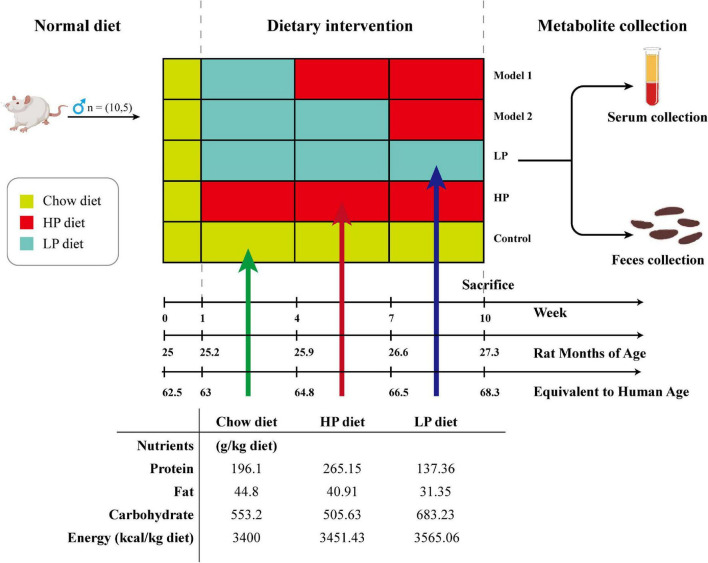
Schematic diagram showing the experimental design, animal grouping, and composition of the experimental diet.

Fifty, 25-month-old, male SD rats (equivalent to human age 62.5 years) were purchased from Ziyuan Lab Animal Ltd. (Hangzhou, China) (36) (animal certification number SCXK-2019-0004). All animal experiments were conducted in accordance with the Animal Ethics Committee of Guangxi University (Approval No. GXU-2022-248). The rats were housed in a room with constant temperature (24 ± 2°C), maintained at a relative humidity of 60 ± 10%, controlled with a 12-h alternating day/night light/dark cycle, and fed ad libitum with the same food and water during the acclimation period. After 1 week of acclimatization, the rats were randomly divided into five groups and received a 10-week dietary intervention (10 rats per group): control group (basal diet), LP diet group (LP diet with a 30% decrease in protein content compared to the basal diet), HP diet group (HP diet with a 30% increase in protein content compared to the basal diet), Model 1 (switched from LP to HP feed at week 4), and Model 2 (switched from LP to HP feed at week 7). Rat feed was purchased from Keao Feed, Ltd. (Beijing, China). LP and HP diets were made from the standard chow as the base, with corn starch, maltodextrin, casein, L-cystine, sucrose, cellulose, lard, mixed vitamin V10037, mixed mineral S10022G, and hydrocholine bitartrate as the configuration ingredients, respectively. To ensure the same daily calorie intake for each group of rats, the weights of the LP and HP feeds were adjusted according to the ratio of total energy to obtain special feeds with total energy consistent with the standard feed. Body weight was measured weekly during the 10-week treatment by means of the ME10402 electronic balance (METTLER TOLEDO Instruments Co., Shanghai, China). After 10 weeks, rat feces were collected. After testing the open-field experiment, all rats were fasted for 12 h, and then were anesthetized with isoflurane and sacrificed by cervical dislocation, and serum and liver tissues were collected. The experimental design, animal grouping, and composition of the experimental diets are shown in Figure 1.

### Open-field experiments

Open-field experiments were performed according to literature (37), and the experiments can assess rats’ spontaneous exploration, locomotor, and anxiolytic abilities in new environments (38). The experimental setup consisted of a 100 × 100 × 40 cm blue medical ABS plastic box and SuperMaze animal behavior analysis software (XR-Xmaze, Shanghai Xinruan Information Technology Co., Shanghai, China) to monitor the behavioral trajectory of the rats. The open-field experiment was conducted after 10 weeks of dietary intervention and the rats were sacrificed on the day after the open-field experiment. The rats were brought into the experimental site 3 h in advance to adapt to the new environment while maintaining low light illumination and silence in the environment. Each rat was placed in the central area of the open field. The average movement speed, number of central area entries, and number of upright hind limbs of rats in the open field were recorded for 5 min. The data were analyzed using the SuperMaze software for statistical and trajectory processing.

### Quantification of liver tissue malondialdehyde, serum total antioxidant capacity, and interleukin-6

The liver was immediately frozen in liquid nitrogen and stored at −80°C. On the day of the assay, the liver tissue was removed and weighed accurately for the assay. Add 4 times the volume of saline by weight and homogenize mechanically in an ice water bath to produce a liver tissue homogenate. MDA in liver tissue and T-AOC in serum were measured using the MDA assay kit (A003-1-1) and the T-AOC assay kit (A015-1-2), respectively (Nanjing Jiancheng Bioengineering Institute, Nanjing, Jiangsu, China), and serum inflammatory factor IL-6 was measured using a rat interleukin-6 ELISA test kit (JL20896) (Shanghai Jianglai Biotechnology Co., Ltd., Shanghai, China).

### Statistical analysis of comprehensive health indicators and quantitative evaluation of health indicators

To comprehensively characterize the effects of protein nutrition on aging of experimental animals, a new determination method termed as the comprehensive quantitative evaluation was established based on the multivariate statistical analysis theory. Principal component analysis (PCA) was used to quantitatively analyze the health status of the rats (39). This study selected five indicators: T-AOC, MDA, IL-6, mean movement speed, and number of upright hind limbs. To eliminate the effect of differences in the data scale of different indicators, the raw data of each indicator were standardized and transformed into dimensionless data. MDA and IL-6 levels, two negative indicators of health status, were normalized, and the control group data were used as a reference to investigate the change in health status. The health statuses of the rats were quantitatively expressed using comprehensive health evaluation indices. The overall evaluation score (*f*_*sum*_) was calculated using the following equation:


(1) $$\begin{array}{l} f_{sum}=\frac{v_{1}}{M}\left(\sum_{=1}^{4}a_{i}X_{i}\right)+\frac{v_{2}}{M}\left(\sum_{=1}^{4}b_{i}X_{i}\right)\\ +\frac{v_{3}}{M}\left(\sum_{=1}^{4}c_{i}X_{i}\right)+\frac{v_{4}}{M}\left(\sum_{=1}^{4}b_{i}X_{i}\right) \end{array}$$

where *X*_*i*_ denotes the standardized values of the indicators for each test group, *a*_*i*_, *b*_*i*_, *c*_*i*_, and *d*_*i*_ are the first, second, third, and fourth principal component values for each group, respectively, *v*_*i*_ is the variance explained by the sample under each principal component, and *M* is the cumulative variance explained by the sample.

### Fecal and serum untargeted metabolomics analysis based on ^1^H-nuclear magnetic resonance

#### Fecal and serum sample preparation for metabolomic analysis

Feces were collected from each group of rats at week 10 by the massage abdomen method and stored in an ultra-low temperature refrigerator at −80°C. Fifty milligrams of feces were collected in a 2 ml centrifuge tube and 500 μl PBS/D_2_O buffer (0.1 M, pH 7.4) containing 10% D_2_O (v/v) and 0.005% sodium 3-(trimethylmethylsilyl)propionate (TSP) (w/v) was added. The samples were vortexed and mixed for 15 s, and then freeze-thawed three times repeatedly with liquid nitrogen. The mixture was homogenized for 2 min in a S10 handheld high-speed homogenizer (Ningbo Xinzhi Biotechnology Co., Ningbo, Zhejiang, China), followed by centrifugation (12,000 *g*, 15 min, 4°C). Supernatants were removed and the extraction procedure was repeated. The supernatant obtained after extraction was collected by centrifugation (12,000 *g* for 20 min at 4°C). The resulting supernatant (550 μl) was added to a 5 mm nuclear magnetic resonance (NMR) tube and mixed well for measurement.

The serum samples obtained were thawed on ice, and 200 μl of serum was aspirated into a 2 ml sterile eppendorf tube and 400 μl of PBS/D_2_O buffer (0.1 M, pH 7.4) containing 15% D_2_O (v/v) was added. The samples were mixed well and centrifuged (12,000 *g*, 20 min, 4°C). The supernatant (550 μl) was slowly aspirated into a 5 mm NMR tube and mixed well for measurement.

#### ^1^H-nuclear magnetic resonance parameters for metabolomics analysis

Prepared rat fecal and serum samples were placed on a Bruker Avance 500 MHz NMR spectrometer (Bruker, Karlsruhe, Germany) with a Prodigy liquid nitrogen cryogenic probe for NMR mapping. The water peaks were suppressed by pre-saturation with a NOESYPR1D pulse sequence (recycle delay − 90°− t_1_ − 90°− t_*m*_ − 90°− acquisition) with the following parameters: number of scans, 64; temperature, 25°C; spectral width, 10,000 Hz; measurement frequency, 500.13 MHz; chirality delay, 2.0 s; sampling points, 65,536 points; sampling time, 3.277 s; fixed echo time, 2 ms; number of cycles, 16.

#### Nuclear magnetic resonance data processing and multivariate statistical analysis

After all tests, data from the fecal and serum samples were imported into MestreNova 14.2 (Mestrelab Research SL, Santiago de Compostela, Spain). An exponential window function with a broadening factor of 0.3 Hz was multiplied before performing the Fourier transform, and the phase was manually adjusted with respect to the baseline. Fecal spectra were calibrated using the methyl resonance at TSP (δ 0.0) as the reference, and serum spectra were calibrated using the methyl resonance at lactate (δ 1.33) as the reference. To eliminate the spurious effect of water suppression, the water peak integral at δ 4.70–5.15 ppm in the fecal spectrum and the water peak integral at δ 4.64–5.10 ppm in the serum spectrum were removed. The region of chemical shift interval δ 0.00–9.00 ppm was integrated into segments with δ 0.002, and the integration results were normalized.

Multivariate statistical analysis of the data was performed using MetaboAnalyst 5.0.^1^ PCA was performed to determine the overall distribution of the samples. Orthogonal partial least squares discriminant analysis (OPLS-DA) was performed to identify differences between the groups, and the stability of the model was ensured by cross-validating the parameters *Q*^2^, *R*^2^, and variance analysis of the cross-validated residuals (CV-ANOVA) (*p* < 0.05). Differential metabolites were screened according to variable projective importance (VIP >1) and independent sample *t*-tests (*p* < 0.05). Differential metabolites with Spearman correlation coefficient | *r*| > 0.4 and *p* < 0.05 were considered metabolites correlated with protein intake. The correlated differential metabolites were then subjected to metabolic pathway analysis based on the Kyoto Encyclopedia of Gene and Genomes (KEGG) database with an impact threshold value >0.08 for metabolic pathways. The results with interaction scores >0.4 were selected using Search Tool for Interactions of Chemicals (STITCH) to construct a metabolic pathway network.

### Statistical analysis

SPSS26.0 software (SPSS Inc., Chicago, IL, USA) was used to statistically analyze the test data. The results are expressed as mean ± SD. Differences in continuous variables, such as body weight, were analyzed using repeated measures ANOVA followed by Tamhane T2 test after performing sphericity test and normality test to ensure the prerequisites for repeated measures ANOVA. The Wilcoxon nonparametric test was used to assess differences in metabolites and biochemical indicators before and after the intervention. Spearman’s correlation analysis was used to examine the relationship between changes in metabolite and protein intake.

## Results

### Effect of protein intake shift on behavioral and physiological parameters in rats

**FIGURE 2:**
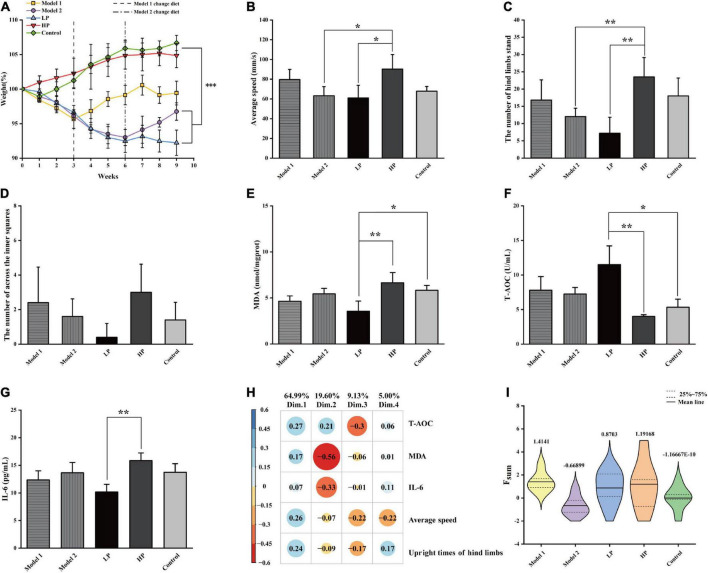
Basic physiological parameters of rats and the comprehensive assessment of aging status. **(A)** Body weight, **(B)** mean movement speed of rats in the open field experiment, **(C)** number of upright hind limbs of rats in the open field experiment, **(D)** number of central region entries of rats in the open field experiment, **(E)** liver MDA level, **(F)** serum T-AOC level, and **(G)** serum inflammatory factor IL-6 level. All values above are expressed as mean ± SD (*n* = 10), *p < 0.05, **p < 0.01, ***p < 0.001. **(H)** Factor loading matrix of the principal components of the health index. **(I)** Violin plot of F_*sum*_ scores of rats in each group.

After dietary interventions, the physiological conditions of the rats were characterized by examining the following seven parameters: body weight, average movement speed during the open-field experiment, number of upright hind limbs, number of central region entries, liver MDA levels, serum T-AOC levels, and serum inflammatory factor IL-6 levels. The body weight results are shown in Figure 2A. The LP diet group lost 7.77% of their body weight over a 9-week period. The HP diet and control groups gained 4.86 and 6.67% of body weight, respectively. The rats in both Model 1 and Model 2 groups regained weight after switching the diet pattern. A 3.76% weight gain was observed in Model 1, and a 3.75% weight gain was observed in Model 2 within 6 weeks after switching the diet. Significant differences were found using repeated measures ANOVA between the LP group and Model 2 and that between HP and control groups (*p* < 0.001). The results indicated that the LP diet effectively reduced the body weight of the aged rats.

The results of the open-field experiment are shown in Figures 2B–D. Unlike the LP group, the HP group showed significant increases of 47.74 and 226.39% in the mean movement speed (Figure 2B) and the number of upright hind limbs (Figure 2C), respectively (*p* < 0.05). In contrast, although there was an increase in the Model 1 group, the difference was insignificant. In addition, the number of upright hind limbs and the number of passes through the center of the open field (Figure 2D) could also indicate the anxiety levels of the experimental animals. The higher the number of upright hind limbs and the higher the number of passes through the open field center, the lower were their anxiety. Although the number of times the rats passed through the center of the open field did not vary significantly among the groups (*p* > 0.05), it was evident that the members of the LP group crossed the center of the open field least, reflecting a higher level of anxiety. Overall, the LP diet increased the anxiety levels of the aging rats, while the HP diet reversed the high anxiety level of rats that were earlier on the LP diet. This also suggests that an LP diet that does not consider the demand pattern is detrimental to the health of the organism.

Malondialdehyde levels in the liver tissue (Figure 2E) were significantly decreased by 46.55% in the LP diet group compared to that in HP diet group (*p* = 0.003) and by 30.18 and 18.32% compared to those in the Model 1 and Model 2 groups, respectively, but the differences were not significant. Serum T-AOC levels (Figure 2F) were significantly higher by 186.78% (*p* = 0.001), and serum IL-6 levels (Figure 2G) were significantly lower by 35.77% (*p* = 0.003) in the LP group than in the HP diet group. Compared with HP diet group, T-AOC levels increased by 94.3 and 80.8%, and IL-6 levels decreased by 22.02 and 13.88% in Model 1 and Model 2, respectively, with no significant differences.

To comprehensively characterize the effects of protein nutrition on aging of experimental animals, the following five indices were selected: average movement speed, number of upright hind limbs, liver MDA levels, serum T-AOC levels, and serum inflammatory factor IL-6 levels. The above indicators were first scored comprehensively and quantitatively using PCA, and four principal components were extracted according to a cumulative variance contribution rate greater than 85%. The contribution of each variable and the factor-loading matrix are shown in Figure 2H, and the cumulative variance contribution rate reached 98.73%. The standardized indicators and factor loading matrix were substituted into Equation 1, and the violin plot of the obtained results is shown in Figure 2I. The results showed that Model 1 had the highest combined quantitative score, followed by the HP and LP groups, while Model 2 had the lowest combined quantitative score. Compared with total LP and HP diets, the switch from an LP to an HP diet produced two extreme divisions. Model 1 with the highest score, indicates that the switch from LP to HP nutrition can help healthy aging in test rats after a certain period of adaptation. Model 2 with the lowest score, indicates that a sudden switch from LP to HP nutrition can negatively impact on the health of the body in a short period (3 weeks).

### Nuclear magnetic resonance metabolic profile of rat feces and serum

**FIGURE 3:**
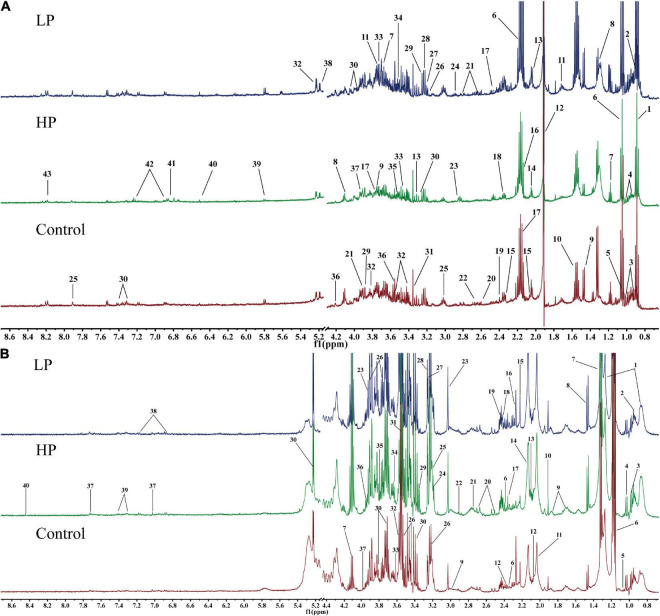
Representative ^1^H-NMR metabolic profiles of rat feces and serum. **(A)** Representative ^1^H-NMR metabolic profiles of rat feces. 1, lipid; 2, butyrate; 3, isoleucine; 4, leucine; 5, valine; 6, propionate; 7, ethanol; 8, lactate; 9, alanine; 10, citrulline; 11, arginine; 12, acetate; 13, proline; 14, N-acetylglucosamine; 15, glutamate; 16, methionine; 17, glutamine; 18, pyruvate; 19, succinate; 20 PUFA; 21, aspartate; 22, dimethylamine; 23, trimethylamine; 24, asparagine; 25, histidine; 26, choline; 27, β-xylose; 28, taurine; 29, betaine; 30, phenylalanine; 31, methanol; 32, α-glucose; 33, β-glucose; 34, inositol; 35, glycine; 36, threonine; 37, glycolate; 38, α-xylose; 39, uracil; 40, fumarate; 41, 3-hydroxyphenyl propionic acid; 42, tyrosine; 43, hypoxanthine. **(B)** Representative ^1^H-NMR metabolic profiles of rat serum. 1, lipid; 2, isoleucine; 3, leucine; 4, valine; 5, isobutyrate; 6, 3-hydroxybutyrate; 7, lactate; 8, alanine; 9, lysine; 10, acetate; 11, N-acetylglucosamine; 12, glutamate; 13, glutamine; 14, O-acetyl glycoprotein; 15, acetone; 16, acetoacetate; 17, pyruvate; 18, succinate 19, carnitine; 20, citrate; 21, dimethylamine; 22, trimethylamine; 23, creatine; 24, choline; 25, phosphorylcholine; 26, β-glucose; 27, trimethylamine oxide; 28, betaine; 29, scyllo-inositol; 30, α-glucose; 31, glycine; 32, threonine; 33, propanetriol; 34, inositol; 35, arginine; 36, 1-methylhistidine; 37, histidine; 38, tyrosine; 39, phenylalanine; 40, formate.

The fecal and serum metabolites of rats were identified based on the chemical shifts and signal diversity of each metabolite, combined with coupling information from the public database BMRB^2^ and 2D NMR profiles from authentic literature (32, 40–42). Forty-three metabolites were detected in rat feces (Figure 3A), and forty were detected in rat serum (Figure 3B). The assignments of major metabolites from fecal and serum samples of rats are shown in Supplementary Tables 1, 2. These metabolites include amino acids (e.g., tyrosine and valine), carbohydrates (e.g., glucose and xylose), intermediates of the TCA cycle (e.g., citrate, fumarate, and succinate), energy metabolites (e.g., pyruvate and lactate), short-chain fatty acids (SCFA) (e.g., acetate and butyrate), ketone bodies (e.g., acetone and 3-hydroxybutyrate), intestinal flora metabolites (e.g., dimethylamine and trimethylamine), glycoproteins (e.g., N-acetylglucosamine), and other substances.

### Differential metabolites in rat fecal and serum samples

Fecal and serum ^1^H-NMR data were analyzed to determine the variability of the metabolites between the groups. The unsupervised PCA scores of feces and serum are shown in Supplementary Figures 1A, 2A, respectively. The p-value of the PERMANOVA test for fecal and serum PCA was less than 0.001, which implied that the intervention of different protein nutritional modalities had a significant effect on the metabolic status of the rats. However, there were groups with non-significant differences in the Adonis paired test for PCA, suggesting that PCA did not completely distinguish between all groups of rats. To achieve maximum separation of fecal and serum metabolites in each group of rats, further analysis was performed using the OPLS-DA model. The OPLS-DA score plots and VIP score rankings for feces and serum are shown in Supplementary Figures 1B–E, 2B–E, respectively. The Q^2^Y of all groups were close to one [*R*^2^ > 0.4, *p*(CV) < 0.05]. The results of the model parameters indicated that the model fit was highly reliable and could effectively separate the fecal and serum metabolites of each group of rats.

Twenty and 13 differential metabolites were screened in feces and serum, respectively, compared to controls based on a combination of VIP values (VIP >1) and the Wilcoxon nonparametric test results (*p* < 0.05). The differential metabolites identified in the feces and serum and their correlation coefficients are shown in Tables 1, 2, respectively. Among fecal metabolites, the levels of amino acids (arginine, histidine, glycine, leucine, tyrosine, glutamate, and threonine), hypoxanthine, and lipids were reduced while lactate, N-acetylglucosamine, and pyruvate levels were elevated in the LP diet group. In contrast, arginine, valine, and acetate levels were increased and the levels of succinate, polyunsaturated fatty acids, uracil, lipids, α-xylose, and N-acetylglucosamine were decreased in the HP diet group. Among the serum metabolites, the levels of alanine, lactate, and pyruvate increased and that of 3-hydroxybutyrate decreased in the LP diet group. Acetate, phenylalanine, tyrosine, and glutamate levels were increased and N-acetylglucosamine levels were decreased in the HP diet group.

**TABLE 1:** Correlation coefficients of fecal differential metabolites between different paired comparison groups.

Metabolites	FC			
	
	Model 1 vs. control	Model 2 vs. control	LP vs. control	HP vs. control
α-Xylose		–0.63536		–1.2312
Acetate				+ 0.31746
Arginine			–0.85319	
Asparagine		–0.60505		
Glutamate		–0.62497	–0.81163	
Glycine	–0.59124	–0.57278	–0.75023	
Histidine	–0.52255	–0.9696	–1.5287	
Hypoxanthine		–0.4194	–0.55583	
Lactate	+ 1.2126	+ 1.2757	+ 0.96242	
Leucine	–0.91305	–1.324	–2.3603	
Lipid	–0.75249	–0.71621	–0.83976	–0.65864
N-acetylglucosamine			+ 0.71973	–0.75979
PUFA	–1.3631			–1.5501
Pyruvate			+ 1.0634	
Succinate	–1.4668			–1.8356
Threonine		–1.3134	–0.9945	
Trimethylamine				–0.35337
Tyrosine			–1.302	
Uracil	–0.83561	–0.75283		–1.1564
Valine				+ 0.66537

FC (fold change) indicates the fold change of metabolites between two groups as the logarithmic value of the mean ratio between two groups with a base of 2; + and − indicate the increase or decrease of metabolite concentration in the previous group, respectively.

**TABLE 2:** Correlation coefficients of serum differential metabolites between different paired comparison groups.

Metabolites	FC			
	
	Model 1 vs. control	Model 2 vs. control	LP vs. control	HP vs. control
3-Hydroxybutyrate	–0.32961	–0.52644	–0.27046	
Acetate	+ 0.3388			+ 0.6747
Alanine	+ 0.29435	+ 0.2173	+ 0.51014	
Choline		+ 0.44545		
Glutamate				+ 0.69206
Glycine	–0.33434	–0.48691		
Isobutyrate	+ 0.75203	+ 1.2462		
Lactate	+ 0.60182	+ 0.83173	+ 0.90002	+ 0.4537
Lysine	+ 0.53865			
N-acetylglucosamine				–0.58918
Phenylalanine		+ 1.3653		+ 0.97413
Pyruvate			+ 0.90236	
Tyrosine				+ 0.65458

FC (fold change) indicates the fold change of metabolites between two groups as the logarithmic value of the mean ratio between two groups with a base of 2; + and − indicate the increase or decrease of metabolite concentration in the previous group, respectively.

**FIGURE 4:**
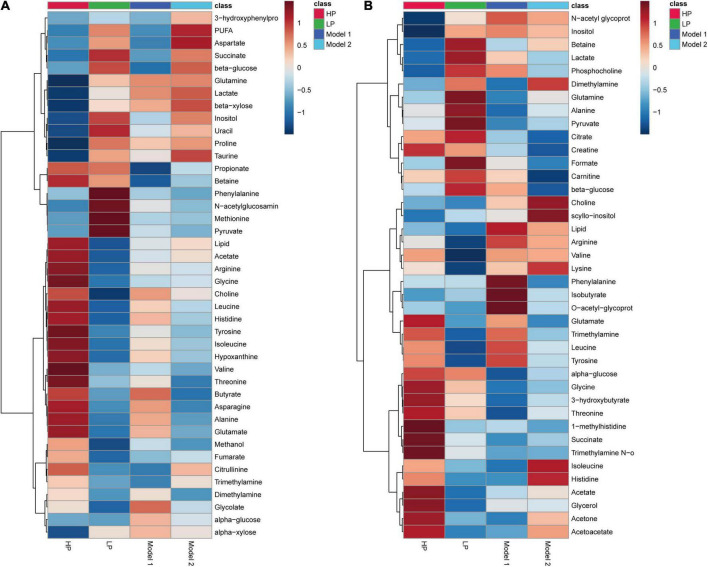
Hierarchical clustering heatmap of metabolite concentrations. **(A)** Hierarchical clustering heatmap of metabolite concentrations in rat feces. **(B)** Hierarchical clustering heatmap of metabolite concentrations in rat serum.

To illustrate the changes in serum and fecal metabolites and the differences in metabolite levels between the groups of rats, we produced metabolite hierarchical clustering heat maps for feces and serum, as shown in Figures 4A,B, respectively. The graphs show that the variation of metabolites in response to diet was greater in feces than in serum. More differential metabolites were identified in feces, and the magnitude of the metabolite changes was greater. The HP diet significantly increased the levels of amino acids and their derivatives and decreased the levels of carbohydrate metabolic intermediates in both feces and serum. The LP diet was found to have an effect opposite to that of the HP diet: the levels of amino acids and their derivatives were reduced, and the levels of carbohydrate metabolic intermediates were increased. Although the only difference between Model 1 and the high-protein group was the 3-week LP diet intervention, the metabolite clustering heat map showed a significant difference between the metabolite concentrations of the two groups.

### Correlation analysis between differential metabolites and protein intake

**FIGURE 5:**
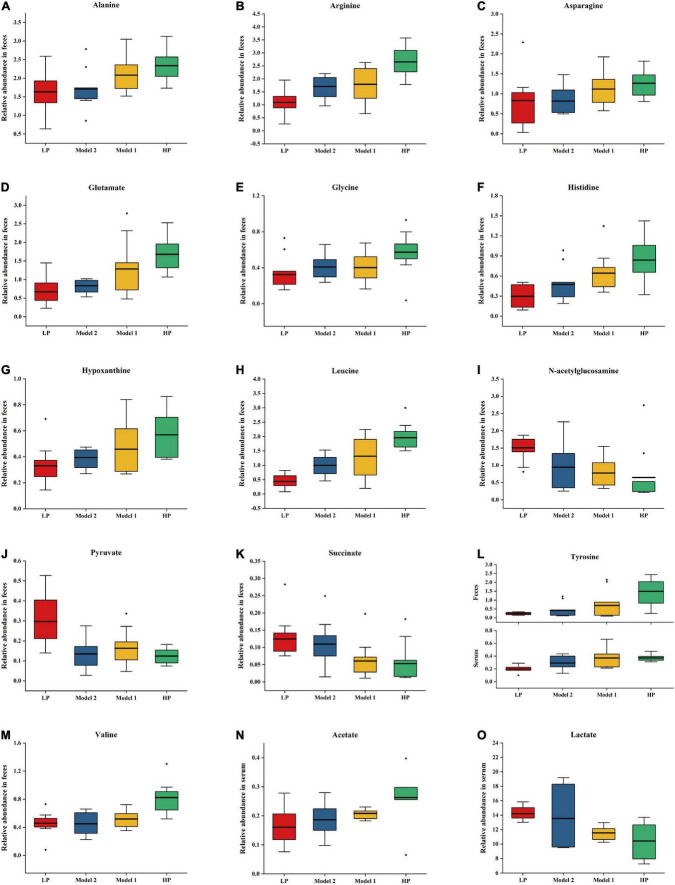
Correlation difference metabolite normalized peak area box plots for Model 1, Model 2, chronic low protein, and chronic high protein diet model groups. **(A)** Alanine, **(B)** arginine, **(C)** asparagine, **(D)** glutamate, **(E)** glycine, **(F)** histidine, **(G)** hypoxanthine, **(H)** leucine, **(I)** N-acetylglucosamine, **(J)** pyruvate, **(K)** succinate, **(L)** tyrosine, **(M)** valine, **(N)** acetate, and **(O)** lactate. Data are expressed as mean ± SD.

The differences in protein intake among the different groups of rats during the dietary intervention led to significant differences in the fecal and serum metabolites between these groups. Therefore, it was necessary to further investigate the correlation between protein intake and differential metabolites in rats using Spearman’s correlation analysis. The selection criteria to identify correlated differential metabolites was set as | *r*| > 0.4 and *p* < 0.05. The normalized peak area box plots of all correlated differential metabolites are shown in Figure 5. The results showed that some essential amino acids (leucine and valine), conditionally essential amino acids (arginine and histidine), and some essential amino acids involved in physiological metabolic regulation (glutamate, tyrosine, alanine, and glycine) in feces were positively correlated with protein intake. In addition, asparagine and hypoxanthine levels were positively correlated with protein intake. In contrast, pyruvate, succinate, and N-acetylglucosamine levels were negatively correlated with protein intake. Serum tyrosine and acetate levels were positively correlated with protein intake, whereas lactate levels were negatively correlated with protein intake.

### Metabolic pathway analysis

**FIGURE 6:**
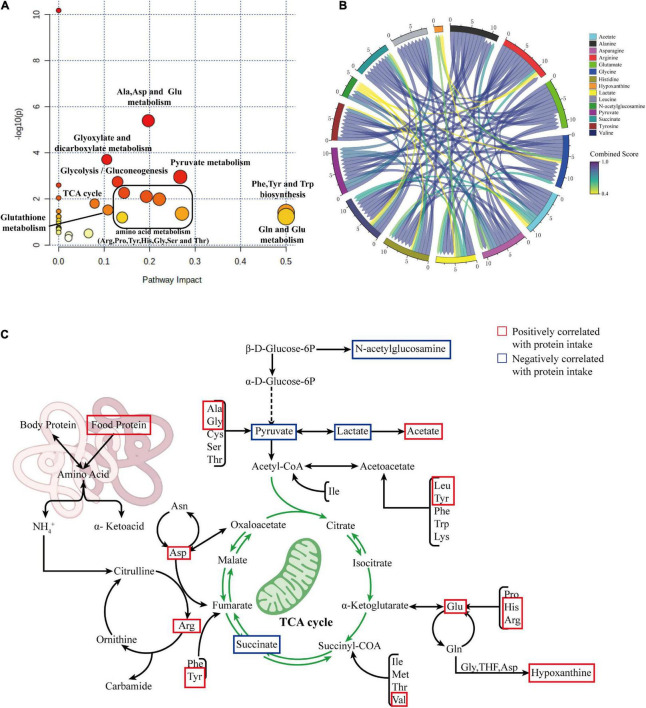
**(A)** Summary plot of pathway analysis of correlated differential metabolites using MetPA. **(B)** Chord diagram showing the relationship between correlated differential metabolites. **(C)** Summary diagram of metabolic pathways associated with protein intake.

To clarify the effects of differential protein intake on biochemical pathways, we further mapped the newly identified differentially correlated metabolites to metabolic pathways using MetPA (Metabolomics Pathway Analysis). The results are shown in Figure 6A. Changes in protein intake mainly affected amino acid synthesis and metabolism, such as arginine biosynthesis; phenylalanine, tyrosine, and tryptophan biosynthesis; histidine metabolism; alanine, aspartate, and glutamate metabolism; arginine and proline metabolism; glycine, serine, and threonine metabolism; and glutathione metabolism. In addition, metabolic pathways related to carbohydrate metabolism, such as glycolysis/gluconeogenesis, glyoxylate, and dicarboxylate metabolism, pyruvate metabolism, and the TCA cycle, were also identified.

To explore the interaction between the correlated differential metabolites in these metabolic pathways, we constructed a metabolic network of correlated differential metabolites using STITCH. The results are shown in Figure 6B. The results indicate that all correlated differential metabolites interact with each other, directly or indirectly. The remaining differential metabolites were closely related, with the exception of hypoxanthine and N-acetylglucosamine. Strong interactions were observed between the amino acids alanine, glycine, arginine, histidine, glutamate, leucine, and asparagine and the metabolites lactate, pyruvate, succinate, and acetate (all interaction scores >0.9). This reflects the strong role of amino acid metabolism in the formation of essential or non-essential amino acids in organisms through amino-acid conversion and the interaction between amino acid and carbohydrate metabolisms. To better understand this situation, we generalized the metabolic pathways disturbed by protein intake. The results are shown in Figure 6C.

## Discussion

Currently, there are different views on protein nutrition requirements in middle-aged and elderly people (43–45). To understand and reveal the underlying mechanisms, this study explored this issue using rat models to mimic the human aging process. The effects of different protein nutritional intakes on health status were examined comprehensively by characterizing aging-related indicators in rat models combined with non-targeted fecal and serum metabolomic changes. A comprehensive quantitative evaluation of behavioral factors, antioxidant capacity, and inflammatory factors in rats showed that a dietary pattern shift from low to high protein had better anti-aging effects after a certain period of adaptation compared to long-term LP or HP diets. The results of the metabolomic analysis also revealed the mechanisms underlying these effects.

### Comprehensive quantitative assessment of aging status

This study found that an LP diet reduces body weight, oxidative stress, and inflammation. The HP diet compensated for the weight loss that occurred with the LP diet and maintained the exercise capacity of the rats. In a study by Mitchell et al., protein restriction was found to improve levels of oxidative stress and inflammation in mice (46). In the study by Mitsuishi et al., weight and exercise capacity were increased in older adults by a HP dietary intervention (47). These results are consistent with the findings in our study. A new quantitative method was established to characterize the health status of rats as scientific and comprehensive as possible. Through PCA, multiple analytical terms were converted into several key generalized indicators. A composite score was obtained using the component scores and variance interpretation rates to comprehensively assess aging status. Model 1 had a higher composite score than the long-term LP and HP diet groups, and Model 2 had the lowest composite score probably because of a 3-week difference in the duration of the HP diet. Walrand et al. found that a brief HP diet for 10 days increased systemic amino acid uptake, but not muscle protein synthesis in older adults (48). Thus, the Model 2 group may have failed to enhance their exercise capacity sufficiently owing to the short duration of the HP intervention, while oxidative stress and inflammation levels were elevated. In a study by Hahn et al., mortality and inflammation levels were reduced in mice subjected to pre-restricted diet followed by free diet compared to those administered free diet for longer periods (49). Protein restriction, a significant cause of anti-aging effect in restricted diets, produced similar results. The members of the Model 1 group, with low protein during the initial period and high protein in the subsequent period, were able to maintain lower levels of oxidative stress and inflammation with enhanced exercise capacity, which would better meet the physiological requirements of the organism for dynamic changes in protein during the natural aging process. As shown in Figure 1, Model 1 and Model 2 showed significant differences in health status when their diets were switched from low to high protein at 64.8 and 66.5 years of age, respectively. During a physical examination at 68.3 years of age, Model 1 scored high and Model 2 scored low. This shows that the health status of an organism at this age is susceptible to changes in protein content in food. These results provide more than sufficient support for the validity of the hypotheses in this study. After approximately 50 years of age, there is a smooth change in protein requirement from lower to higher rates that can be beneficial to health. The critical point is estimated to be around 65 years, and a smooth transition is required as drastic changes may be detrimental to health.

It is particularly important to note that this study found that in the period equivalent to the human age of 62.5–68.3 years, the most beneficial way of protein nutrition for a long and healthy life is a gradual change in demand from low to high (Model 1), and simple low and high protein nutrition are not as effective as this. In the reality of several countries, people in that period have just retired, are financially well-off, still in good health, and have a simple concept of health care, among other reasons, contributing to the excessive protein intake of many people. This will harm many issues such as their pre-retirement performance, healthy longevity, and health care expenses. Therefore, it is considered necessary to further explore protein nutrition in people around the age of menopause to 70 years based on this finding. This is not only a scientific issue, but should also be a far-reaching social issue that needs to be addressed urgently.

### Non-targeted fecal and serum metabolomics and metabolites

Next, non-targeted fecal and serum metabolomics were performed in this study to reveal the nutritional effects and mechanisms of action of proteins. Non-targeted fecal and serum metabolomic analyses identified 15 differential metabolites relevant to protein intake. The changes in fecal and serum metabolomic profiles were mainly enriched in amino acid and carbohydrate metabolism. The intervention with an LP diet in the early years can improve the metabolic profile if followed up with an HP diet in the later years and thus positively impact health according to the appropriately chosen period. A middle-aged LP followed by an elderly HP diet can enhance amino acid metabolism while maintaining carbohydrate metabolism, thus preventing mitochondrial dysfunction and frailty. This metabolomic analysis provides a theoretical basis for the shift from a low-protein to a high-protein nutritional approach in middle-aged and older adults from a metabolic perspective.

#### Amino acid metabolism

As protein intake increased, fecal levels of leucine, valine, arginine, histidine, glutamate, tyrosine, and glycine increased, positively correlating with protein intake. Contents of stool samples are directly related to dietary patterns and health status, and changes in fecal metabolites can reflect potential mechanisms in the colon-systemic axis of food metabolism (50). Studies have shown that increased levels of amino acids in fecal metabolism increase the risk of developing diseases such as obesity and diabetes during the aging process in middle-aged adults. Levels of branched-chain amino acids, such as leucine and valine, are associated with metabolic syndromes, such as obesity and diabetes mellitus (51). Significant increases in glutamate, leucine, and valine levels have been found in stools of patients (52). Studies in diabetic mice have also found a significant upregulation of amino acids among fecal metabolites (phenylalanine, a precursor of tyrosine metabolism, and aspartate, a precursor of glutamate metabolism) (53). Therefore, increased levels of branched-chain amino acids, glutamate, and tyrosine in feces can harm the health of elderly individuals. Correlation analysis found that the levels of the relevant amino acids decreased in both Model 1 and Model 2 groups after different LP intervention durations in the first period. This implies that, by intervening with an LP diet upfront, the excessive elevation of amino acid levels in the gut can be limited at a later stage, thus reducing the negative effects of an HP diet on the health of the elderly population in terms of metabolic diseases.

In addition, we found an increase in tyrosine, glutamate, and phenylalanine levels in the serum following HP intervention. Serum metabolites indicate nutrient bioavailability and reflect individual metabolic changes (54). Glutamate is central to the processing of the daily protein load, and arginine, ornithine, proline, histidine, and glutamine require conversion to glutamate to enter the metabolic cycle with significant metabolic versatility (55). Glutamate can increase the synthesis of glutamine, which serves as an important component of muscle proteins and contributes to muscle repair and strengthening (56). Studies in adults, pigs, and preterm infants have found that glutamate is metabolized primarily in the gastrointestinal tract and that serum glutamate concentrations return to normal within 2 h, even when high doses of monosodium glutamate are ingested (57). However, during HP dietary intervention in elderly men, the plasma metabolic profile showed significant increase in glutamate levels (58). This may imply that as the body ages, the ability to catabolize glutamate decreases, affecting multiple metabolic pathways, such as gluconeogenesis, carbohydrate metabolism, and glutathione metabolism. Additionally, frailty in the elderly population includes not only a decrease in motor capacity but also a decrease in cognitive ability (59). Tyrosine and phenylalanine play multiple biological roles. Increased protein intake has been shown to increase tyrosine and phenylalanine concentrations in the brain, stimulating the production of catecholamines, which function in active excitatory neurons (60). This process is influenced by the availability of amino acids in the blood. The correlation analysis results from our study showed that tyrosine serum metabolites were positively correlated with protein intake. Serum tyrosine levels in the Model 1 and Model 2 groups increased after later HP intervention, suggesting that the elevated protein intake in the later period enhanced serum amino acid metabolism, which may play a positive role in maintaining young muscle synthesis and cognitive ability in the elderly.

#### Carbohydrate metabolism

Pyruvate, succinate, and lactate are important components of the carbohydrate metabolism. Among fecal metabolites, pyruvate and succinate levels were negatively correlated with protein intake, and among serum metabolites, lactate level was negatively correlated with protein intake. A significant increase in pyruvate level was found in the fecal and serum metabolites of the LP group. Because an isocaloric dietary intervention was used, the increase in pyruvate, succinate, and lactate levels in the LP group was more likely due to the increased proportion of carbohydrates in the diet. Accelerated TCA cycling following high-carbohydrate intake has been associated with various diseases in the elderly, such as cancer and atherosclerosis (61). However, other reports have demonstrated that reduced pyruvate metabolism and TCA cycle capacity during aging lead to mitochondrial dysfunction (62). It also interferes with ATP production, impairs cellular function, and leads to tissue dysfunction (63). In addition, some inflammatory responses of microglia are associated with dysregulated glucose metabolism (64). Correlation analysis revealed that both Model 1 and Model 2 groups showed a significant increase in pyruvate, succinate, and lactate levels after HP diet following a pre-LP intervention compared to HP group levels. Therefore, it can be hypothesized that a pre-LP dietary intervention can contribute to healthy aging in the elderly population by improving the dysregulation of glucose metabolism and maintaining an optimal level of glucose metabolism after HP dietary intervention.

#### Intestinal fatty acid metabolism and other metabolic pathways

A significant increase in acetate level was observed in feces of rats following an HP diet, and a significant increase in lactate level was observed in those following an LP diet. It is possible that changes in the proportion of proteins in the diet altered the composition and abundance of intestinal flora. Acetate and lactate in feces are mainly produced by the fermentation of undigested carbohydrates by intestinal microorganisms (65). Acetate accounts for the highest percentage of SCFA produced by the intestinal flora and can regulate intestinal pH and maintain intestinal stability (66). Human and animal models following HP diets have demonstrated an elevation in SCFA (67, 68). HP diets cause an increase in the proportion of proteins that remain incompletely digested after passing through the small intestine, allowing microorganisms to produce more SCFA and other protein fermentation products, mainly using peptides as a carbon source (69). These SCFA and protein fermentation products can regulate protein homeostasis in muscle cells and promote muscle anabolism (70), implying that protein intake can counteract muscle loss in the elderly through the intestinal flora. In the current study, lactate levels in fecal metabolites were significantly increased not only in the LP diet group but also in Model 1 and Model 2 groups. Lactate in the gut is a pro- and anti-inflammatory regulator that reduces inflammation by decreasing damage to cells in the intestinal wall and inhibiting the release of pro-inflammatory cytokines, such as IL-6 (71). This may be an underlying mechanism by which an LP diet upfront and an HP diet later can positively affect intestinal health and reduce inflammation in the elderly population by maintaining intestinal wall integrity.

We also observed a positive correlation between hypoxanthine levels in feces and protein intake. Due to increased protein intake, the amino acids involved in de novo synthesis increased. Gut microbes have an enhanced ability to synthesize purines de novo, thus producing more hypoxanthine (72). The lack of significant increase in hypoxanthine levels in the serum metabolites following an HP diet may be due to the conversion of hypoxanthine to uric acid, prompted by the high activity of xanthine oxidoreductase in rats (73). Many studies have shown that an HP diet leads to an increase in uric acid levels and increases the risk of gout and hyperuricemia in the elderly population (58, 74), which is caused by the abnormal metabolism of uric acid due to decreased renal function in the elderly population (75). This implies that elderly people with renal insufficiency, diabetes, and gout attacks must be more cautious when adopting an HP diet. Compared to the fecal hypoxanthine levels in the HP group, they were reduced in both the Model 1 and Model 2 groups, and the initial LP diet had inhibited excessive uric acid production. This suggests that introducing an LP diet in the first phase and an HP diet in the second phase have a wider range of applicability and should be a more scientific and rational way to promote a long and healthy life than introducing a long-term HP diet to prevent muscle loss in old age.

This study has some limitations: (1) A non-targeted ^1^H-NMR method for metabolomic analysis was used. To gain a more comprehensive understanding of the overall metabolic process of initial LP diet followed by an HP diet, new methods such as proteomics and lipid metabolomics can be applied in future to investigate the mechanism of anti-aging and effects of such a diet. (2) The indices covered by the comprehensive quantitative evaluation in this study may not fully represent the health status of the organism. In future studies, it will be necessary to include more indices for testing. If the parameters of histological analysis are unified into the system of comprehensive quantitative scoring, more representative data information can be obtained. (3) Only male rats were selected for study in our experiments. Sex differences in aging occur in many animal species, including sex differences in lifespan, onset and progression of age-related decline, and physiological and molecular markers of aging (76). And both the immune system and the aging process in adult mammals are sexually dimorphic, suggesting that aging studies should be stratified according to sex (77, 78). Therefore, to make this model of protein diet based on age-differentiated nutritional strategies applicable to a broader population, it is necessary to include the sex system in future studies. (4) This study was conducted in rats. In subsequent experiments, it is necessary to recruit middle-aged and elderly volunteers for dietary intervention experiments with increased group sizes, decreased intensity of protein level changes, and extended intervention duration. Thus, we can further investigate the effect of protein diets on different ages, nutrition strategies, and the health status of middle-aged and elderly people to provide scientific and technological support for human health and longevity.

## Conclusion

Comprehensive quantitative scoring of the overall health of the rats and ^1^H-NMR-based metabolomic analysis showed that the amount of protein intake, the timing of intake, and the duration of dietary intervention are key factors affecting fitness. Protein supplementation in food should closely match the increased physiological demand for protein that occurs with natural aging of the organism, and a sudden increase in protein intake is detrimental to the health of the organism. Mechanistic studies have found that LP dietary interventions in the middle-age can compensate to some extent for the damage caused by an HP diet in old age, in terms of oxidative damage and inflammation. The later HP diet then helps compensate for the lack of muscle synthesis capacity of the earlier LP diet, thus reducing the risk of protein malnutrition in the elderly population. Fecal and serum metabolomic analyses identified 43 and 40 metabolites, respectively, among which 20 and 13 were identified as differential metabolites, respectively. Eight amino acids and two other substances in feces were significantly positively associated with protein intake, and three substances, including pyruvate, were significantly negatively associated. Serum tyrosine and lactate levels were positively correlated with protein intake, whereas acetate levels were negatively correlated. Metabolic pathway analysis revealed that the LP diet activated carbohydrate metabolism and accelerated the TCA cycle. The HP diet increased energy metabolism more than amino acid metabolism, thus preventing muscle loss in older age groups due to reduced protein synthesis capacity. In summary, the results of combined quantitative scoring and metabolomics analysis supports the hypothesis from different perspectives. After about 50 years of age, there is a change in the nutritional requirements of protein for health from a lower to a higher rate. This study brings together different opinions on protein nutrition strategies and provides an important reference to construct a scientific and rational approach to protein nutrition. This will be significant in helping middle-aged and elderly populations to improve their physical fitness and achieve healthy aging.

## Data availability statement

The original contributions presented in this study are included in the article/Supplementary material, further inquiries can be directed to the corresponding author.

## Ethics statement

The animal study was reviewed and approved by the Animal Ethics Committee of Guangxi University.

## Author contributions

QL, WZ, and YZ: experimental idea conception and experimental design. FS, YL, FZ, XZ, and JL: experimental reagents and materials preparation and experimental investigation. RL and YS: data organization and visualization of experimental results. WZ: full manuscript writing. QL: full review and revision of the manuscript. All authors reviewed the manuscript and approved the final manuscript.

## Footnotes

1
https://www.metaboanalyst.ca/

2
http://www.bmrb.wisc.edu

## References

[1] Wang D Ye J Shi R Zhao B Liu Z Lin W Dietary protein and amino acid restriction: Roles in metabolic health and aging-related diseases. *Free Radic Biol Med.* (2022) 178:226–42. 10.1016/j.freeradbiomed.2021.12.009 34890767

[2] Bhupathiraju SN Hu FB. Epidemiology of obesity and diabetes and their cardiovascular complications. *Circ Res.* (2016) 118:1723–35. 10.1161/CIRCRESAHA.115.306825 27230638 PMC4887150

[3] Elias MF Elias PK Sullivan LM Wolf PA D’Agostino RB. Obesity, diabetes and cognitive deficit: The Framingham Heart Study. *Neurobiol Aging.* (2005) 26(Suppl 1):11–6. 10.1016/j.neurobiolaging.2005.08.019 16223549

[4] Wolf PA Beiser A Elias MF Au R Vasan RS Seshadri S. Relation of obesity to cognitive function: Importance of central obesity and synergistic influence of concomitant hypertension. The Framingham Heart Study. *Curr Alzheimer Res.* (2007) 4:111–6. 10.2174/156720507780362263 17430232

[5] Grandison RC Piper MD Partridge L. Amino-acid imbalance explains extension of lifespan by dietary restriction in Drosophila. *Nature.* (2009) 462:1061–4. 10.1038/nature08619 19956092 PMC2798000

[6] Solon-Biet SM McMahon AC Ballard J Ruohonen K Wu LE Cogger VC The ratio of macronutrients, not caloric intake, dictates cardiometabolic health, aging, and longevity in ad libitum-fed mice. *Cell Metab.* (2020) 31:654. 10.1016/j.cmet.2020.01.010 32130886

[7] Mair W Piper MD Partridge L. Calories do not explain extension of life span by dietary restriction in Drosophila. *PLoS Biol.* (2005) 3:e223. 10.1371/journal.pbio.0030223 16000018 PMC1140680

[8] Longo VD Antebi A Bartke A Barzilai N Brown-Borg HM Caruso C Interventions to slow aging in humans: Are we ready? *Aging Cell.* (2015) 14:497–510. 10.1111/acel.12338 25902704 PMC4531065

[9] Fontana L Cummings NE Arriola AS Neuman JC Kasza I Schmidt BA Decreased consumption of Branched-Chain amino acids improves metabolic health. *Cell Rep.* (2016) 16:520–30. 10.1016/j.celrep.2016.05.092 27346343 PMC4947548

[10] Cummings NE Williams EM Kasza I Konon EN Schaid MD Schmidt BA Restoration of metabolic health by decreased consumption of branched-chain amino acids. *J Physiol.* (2018) 596:623–45. 10.1113/JP275075 29266268 PMC5813603

[11] Mu WC VanHoosier E Elks CM Grant RW. Long-Term effects of dietary protein and Branched-Chain amino acids on metabolism and inflammation in mice. *Nutrients.* (2018) 10:918. 10.3390/nu10070918 30021962 PMC6073443

[12] Pietri P Stefanadis C. Cardiovascular aging and longevity: JACC State-of-the-Art review. *J Am Coll Cardiol.* (2021) 77:189–204. 10.1016/j.jacc.2020.11.023 33446313

[13] Sedzikowska A Szablewski L. Insulin and insulin resistance in Alzheimer’s disease. *Int J Mol Sci.* (2021) 22:9987. 10.3390/ijms22189987 34576151 PMC8472298

[14] Kouvari M D’Cunha NM Travica N Sergi D Zec M Marx W Metabolic syndrome, cognitive impairment and the role of diet: A narrative review. *Nutrients.* (2022) 14:333. 10.3390/nu14020333 35057514 PMC8780484

[15] Rodriguez-Manas L Angulo J Carnicero JA El AM Garcia-Garcia FJ Sinclair AJ. Dual effects of insulin resistance on mortality and function in non-diabetic older adults: Findings from the Toledo Study of Healthy Aging. *Geroscience.* (2022) 44:1095–108. 10.1007/s11357-021-00384-4 34075557 PMC9135930

[16] Hahn O Gronke S Stubbs TM Ficz G Hendrich O Krueger F Dietary restriction protects from age-associated DNA methylation and induces epigenetic reprogramming of lipid metabolism. *Genome Biol.* (2017) 18:56. 10.1186/s13059-017-1187-1 28351387 PMC5370449

[17] Pignatti C D’Adamo S Stefanelli C Flamigni F Cetrullo S. Nutrients and pathways that regulate health span and life span. *Geriatrics (Basel).* (2020) 5:95. 10.3390/geriatrics5040095 33228041 PMC7709628

[18] Hanjani NA Vafa M. Protein restriction, epigenetic diet, intermittent fasting as new approaches for preventing age-associated diseases. *Int J Prev Med.* (2018) 9:58. 10.4103/ijpvm.IJPVM_397_16 PMC6036773 30050669

[19] Brunet A Rando TA. Interaction between epigenetic and metabolism in aging stem cells. *Curr Opin Cell Biol.* (2017) 45:1–7. 10.1016/j.ceb.2016.12.009 28129586 PMC5482778

[20] Gavrilova NS Gavrilov LA. Comments on dietary restriction, Okinawa diet and longevity. *Gerontology.* (2012) 58:224–6. 10.1159/000329894 21893946 PMC3362219

[21] Han K Ma J Dou J Hao D Zhu W Yu X A clinical trial of the effects of a dietary pattern on health metrics and fecal metabolites in volunteers with risk of cardiovascular disease. *Front Nutr.* (2022) 9:853365. 10.3389/fnut.2022.853365 35619960 PMC9128613

[22] Wolfe RR. The role of dietary protein in optimizing muscle mass, function and health outcomes in older individuals. *Br J Nutr.* (2012) 108(Suppl 2):S88–93. 10.1017/S0007114512002590 23107552

[23] Arthur ST Cooley ID. The effect of physiological stimuli on sarcopenia; Impact of Notch and Wnt signaling on impaired aged skeletal muscle repair. *Int J Biol Sci.* (2012) 8:731–60. 10.7150/ijbs.4262 22701343 PMC3371570

[24] Green CL Pak HH Richardson NE Flores V Yu D Tomasiewicz JL Sex and genetic background define the metabolic, physiologic, and molecular response to protein restriction. *Cell Metab.* (2022) 34:209–26. 10.1016/j.cmet.2021.12.018 35108511 PMC8865085

[25] Levine ME Suarez JA Brandhorst S Balasubramanian P Cheng CW Madia F Low protein intake is associated with a major reduction in IGF-1, cancer, and overall mortality in the 65 and younger but not older population. *Cell Metab.* (2014) 19:407–17. 10.1016/j.cmet.2014.02.006 24606898 PMC3988204

[26] Timmons JF Hone M Cogan KE Duffy O Egan B. Increased leg strength after concurrent aerobic and resistance exercise training in older adults is augmented by a whole Food-Based high protein diet intervention. *Front Sports Act Living.* (2021) 3:653962. 10.3389/fspor.2021.653962 33842881 PMC8034230

[27] Kim HK Chijiki H Fukazawa M Okubo J Ozaki M Nanba T. Supplementation of protein at breakfast rather than at dinner and lunch is effective on skeletal muscle mass in older adults. *Front Nutr.* (2021) 8:797004. 10.3389/fnut.2021.797004 34993224 PMC8724572

[28] Halbesma N Bakker SJ Jansen DF Stolk RP De Zeeuw D De Jong PE High protein intake associates with cardiovascular events but not with loss of renal function. *J Am Soc Nephrol.* (2009) 20:1797–804. 10.1681/ASN.2008060649 19443643 PMC2723984

[29] Sluijs I Beulens JW van der ADL Spijkerman AM Grobbee DE van der Schouw YT. Dietary intake of total, animal, and vegetable protein and risk of type 2 diabetes in the European Prospective Investigation into Cancer and Nutrition (EPIC)-NL study. *Diabetes Care.* (2010) 33:43–8. 10.2337/dc09-1321 19825820 PMC2797984

[30] Snelson M Clarke RE Nguyen TV Penfold SA Forbes JM Tan SM Long term high protein diet feeding alters the microbiome and increases intestinal permeability, systemic inflammation and kidney injury in mice. *Mol Nutr Food Res.* (2021) 65:e2000851. 10.1002/mnfr.202000851 33547877

[31] Fontana L Adelaiye RM Rastelli AL Miles KM Ciamporcero E Longo VD Dietary protein restriction inhibits tumor growth in human xenograft models. *Oncotarget.* (2013) 4:2451–61. 10.18632/oncotarget.1586 24353195 PMC3926840

[32] Zhao F Gao L Qin X Du G Zhou Y. The intervention effect of licorice in d-galactose induced aging rats by regulating the taurine metabolic pathway. *Food Funct.* (2018) 9:4814–21. 10.1039/c8fo00740c 30131986

[33] Cui CM Wang CS Han SS Yu DY Zhu L Jiang P. Impact of a long-term high-fructose diet on systemic metabolic profiles of mice. *FASEB Bioadv.* (2022) 4:560–72. 10.1096/fba.2021-00152 35949511 PMC9353457

[34] Chak CM Lacruz ME Adam J Brandmaier S Covic M Huang J Ageing investigation using Two-Time-Point metabolomics data from KORA and CARLA studies. *Metabolites.* (2019) 9:44. 10.3390/metabo9030044 30841604 PMC6468431

[35] Deelen J Kettunen J Fischer K van der Spek A Trompet S Kastenmuller G A metabolic profile of all-cause mortality risk identified in an observational study of 44,168 individuals. *Nat Commun.* (2019) 10:3346. 10.1038/s41467-019-11311-9 31431621 PMC6702196

[36] Andreollo NA Santos EF Araujo MR Lopes LR. Rat’s age versus human’s age: What is the relationship? *Arq Bras Cir Dig.* (2012) 25:49–51. 10.1590/s0102-67202012000100011 22569979

[37] Seibenhener ML Wooten MC. Use of the open field maze to measure locomotor and anxiety-like behavior in mice. *Jove-J Vis Exp.* (2015) 6:e52434. 10.3791/52434 25742564 PMC4354627

[38] Harro J. Animals, anxiety, and anxiety disorders: How to measure anxiety in rodents and why. *Behav Brain Res.* (2018) 352:81–93. 10.1016/j.bbr.2017.10.016 29050798

[39] Mei LH Zheng WX Zhao ZT Meng N Zhang QR Zhu WJ A Pilot Study of the Effect of *Lactobacillus casei* obtained from long-lived elderly on blood biochemical, oxidative, and inflammatory markers, and on gut microbiota in young volunteers. *Nutrients.* (2021) 13:3891. 10.3390/nu13113891 34836153 PMC8622130

[40] Liu X Zhao D Zhao S Li Z Wang Y Qin X. Deciphering the correlations between aging and constipation by metabolomics and network pharmacology. *Aging.* (2021) 13:3798–818. 10.18632/aging.202340 33428599 PMC7906210

[41] Miyaguti N Stanisic D Oliveira S Dos SG Manhe BS Tasic L Serum and muscle (1)H NMR-Based metabolomics profiles reveal metabolic changes influenced by a maternal Leucine-Rich diet in Tumor-Bearing adult offspring rats. *Nutrients* (2020) 12:2106. 10.3390/nu12072106 32708621 PMC7400806

[42] Zhao D Liu X Zhao S Li Z Qin X. 1H NMR-Based fecal metabolomics reveals changes in gastrointestinal function of aging rats induced by d-Galactose. *Rejuvenation Res.* (2021) 24:86–96. 10.1089/rej.2020.2352 32847490

[43] Traylor DA Gorissen SHM Phillips SM. Perspective: Protein requirements and optimal intakes in aging: Are we ready to recommend more than the recommended daily allowance? *Adv Nutr.* (2018) 9:171–82. 10.1093/advances/nmy003 29635313 PMC5952928

[44] Deutz NEP Bauer JM Barazzoni R Biolo G Boirie Y Bosy-Westphal A Protein intake and exercise for optimal muscle function with aging: Recommendations from the ESPEN Expert Group. *Clin Nutr.* (2014) 33:929–36. 10.1016/j.clnu.2014.04.007 24814383 PMC4208946

[45] Longo VD Anderson RM. Nutrition, longevity and disease: From molecular mechanisms to interventions. *Cell.* (2022) 185:1455–70. 10.1016/j.cell.2022.04.002 35487190 PMC9089818

[46] Mitchell SE Delville C Konstantopedos P Hurst J Derous D Green C The effects of graded levels of calorie restriction: II. Impact of short term calorie and protein restriction on circulating hormone levels, glucose homeostasis and oxidative stress in male C57BL/6 mice. *Oncotarget.* (2015) 6:23213–37. 10.18632/oncotarget.4003 26061745 PMC4695113

[47] Mitsuishi M Miyashita K Muraki A Tamaki M Tanaka K Itoh H. Dietary protein decreases exercise endurance through rapamycin-sensitive suppression of muscle mitochondria. *Am J Physiol Endocrinol Metab.* (2013) 305:E776–84. 10.1152/ajpendo.00145.2013 23880314

[48] Walrand S Short KR Bigelow ML Sweatt AJ Hutson SM Nair KS. Functional impact of high protein intake on healthy elderly people. *Am J Physiol Endocrinol Metab.* (2008) 295:E921–8. 10.1152/ajpendo.90536.2008 18697911 PMC2575899

[49] Hahn O Drews LF Nguyen A Tatsuta T Gkioni L Hendrich O A nutritional memory effect counteracts benefits of dietary restriction in old mice. *Nat Metab.* (2019) 1:1059–73. 10.1038/s42255-019-0121-0 31742247 PMC6861129

[50] Vanden BJ Marzorati M Laukens D Vanhaecke L. Validated high resolution mass Spectrometry-Based approach for metabolomic fingerprinting of the human gut phenotype. *Anal Chem.* (2015) 87:10927–34. 10.1021/acs.analchem.5b02688 26451617

[51] Siddik M Shin AC. Recent progress on Branched-Chain amino acids in obesity, diabetes, and beyond. *Endocrinol Metab (Seoul).* (2019) 34:234–46. 10.3803/EnM.2019.34.3.234 31565875 PMC6769348

[52] Palmer ND Stevens RD Antinozzi PA Anderson A Bergman RN Wagenknecht LE Metabolomic profile associated with insulin resistance and conversion to diabetes in the Insulin Resistance Atherosclerosis Study. *J Clin Endocrinol Metab.* (2015) 100:E463–8. 10.1210/jc.2014-2357 25423564 PMC4333040

[53] Zhu Y Cong W Shen L Wei H Wang Y Wang L Fecal metabonomic study of a polysaccharide, MDG-1 from *Ophiopogon japonicus* on diabetic mice based on gas chromatography/time-of-flight mass spectrometry (GC TOF/MS). *Mol Biosyst.* (2014) 10:304–12. 10.1039/c3mb70392d 24292023

[54] Ulaszewska MM Weinert CH Trimigno A Portmann R Andres LC Badertscher R Nutrimetabolomics: An integrative action for metabolomic analyses in human nutritional studies. *Mol Nutr Food Res.* (2019) 63:e1800384. 10.1002/mnfr.201800384 30176196

[55] Brosnan JT. Glutamate, at the interface between amino acid and carbohydrate metabolism. *J Nutr.* (2000) 130:988S–90S. 10.1093/jn/130.4.988S 10736367

[56] Meynial-Denis D. Glutamine metabolism in advanced age. *Nutr Rev.* (2016) 74:225–36. 10.1093/nutrit/nuv052 26936258 PMC4892310

[57] Tome D. The roles of dietary glutamate in the intestine. *Ann Nutr Metab.* (2018) 73(Suppl 5):15–20. 10.1159/000494777 30508814

[58] Durainayagam B Mitchell CJ Milan AM Zeng N Sharma P Mitchell SM Impact of a high protein intake on the plasma metabolome in elderly males: 10 Week Randomized Dietary Intervention. *Front Nutr.* (2019) 6:180. 10.3389/fnut.2019.00180 31867339 PMC6910071

[59] Hamezah HS Durani LW Ibrahim NF Yanagisawa D Kato T Shiino A Volumetric changes in the aging rat brain and its impact on cognitive and locomotor functions. *Exp Gerontol.* (2017) 99:69–79. 10.1016/j.exger.2017.09.008 28918364

[60] Fernstrom JD Fernstrom MH. Tyrosine, phenylalanine, and catecholamine synthesis and function in the brain. *J Nutr.* (2007) 137:1539S–47S. 10.1093/jn/137.6.1539S 17513421

[61] Matsuda M Shimomura I. Increased oxidative stress in obesity: Implications for metabolic syndrome, diabetes, hypertension, dyslipidemia, atherosclerosis, and cancer. *Obes Res Clin Pract.* (2013) 7:e330–41. 10.1016/j.orcp.2013.05.004 24455761

[62] Baccolo G Stamerra G Coppola DP Orlandi I Vai M. Mitochondrial metabolism and aging in yeast. *Int Rev Cell Mol Biol.* (2018) 340:1–33. 10.1016/bs.ircmb.2018.05.001 30072089

[63] Jendrach M Pohl S Voth M Kowald A Hammerstein P Bereiter-Hahn J. Morpho-dynamic changes of mitochondria during ageing of human endothelial cells. *Mech Ageing Dev.* (2005) 126:813–21. 10.1016/j.mad.2005.03.002 15888336

[64] Baik SH Kang S Lee W Choi H Chung S Kim JI A breakdown in metabolic reprogramming causes microglia dysfunction in Alzheimer’s disease. *Cell Metab.* (2019) 30:493–507. 10.1016/j.cmet.2019.06.005 31257151

[65] Yao J Chen Y Xu M. The critical role of short-chain fatty acids in health and disease: A subtle focus on cardiovascular disease-NLRP3 inflammasome-angiogenesis axis. *Clin Immunol.* (2022) 238:109013. 10.1016/j.clim.2022.109013 35436628

[66] Louis P Scott KP Duncan SH Flint HJ. Understanding the effects of diet on bacterial metabolism in the large intestine. *J Appl Microbiol.* (2007) 102:1197–208. 10.1111/j.1365-2672.2007.03322.x 17448155

[67] Rios-Covian D Gonzalez S Nogacka AM Arboleya S Salazar N Gueimonde M An overview on fecal branched Short-Chain fatty acids along human life and as related with body mass index: Associated dietary and anthropometric factors. *Front Microbiol.* (2020) 11:973. 10.3389/fmicb.2020.00973 32547507 PMC7271748

[68] Yu H Guo Z Shen S Shan W. Effects of taurine on gut microbiota and metabolism in mice. *Amino Acids.* (2016) 48:1601–17. 10.1007/s00726-016-2219-y 27026373

[69] Aguirre M Eck A Koenen ME Savelkoul P Budding AE Venema K. Diet drives quick changes in the metabolic activity and composition of human gut microbiota in a validated in vitro gut model. *Res Microbiol.* (2016) 167:114–25. 10.1016/j.resmic.2015.09.006 26499094

[70] Prokopidis K Cervo MM Gandham A Scott D. Impact of protein intake in older adults with sarcopenia and obesity: A gut microbiota perspective. *Nutrients.* (2020) 12:2285. 10.3390/nu12082285 32751533 PMC7468805

[71] Parada VD De la Fuente MK Landskron G Gonzalez MJ Quera R Dijkstra G Short chain fatty acids (SCFAs)-Mediated gut epithelial and immune regulation and its relevance for inflammatory bowel diseases. *Front Immunol.* (2019) 10:277. 10.3389/fimmu.2019.00277 30915065 PMC6421268

[72] Hou Y Hu S Li X He W Wu G. Amino acid metabolism in the liver: Nutritional and physiological significance. *Adv Exp Med Biol.* (2020) 1265:21–37. 10.1007/978-3-030-45328-2_2 32761568

[73] Furuhashi M. New insights into purine metabolism in metabolic diseases: Role of xanthine oxidoreductase activity. *Am J Physiol Endocrinol Metab.* (2020) 319:E827–34. 10.1152/ajpendo.00378.2020 32893671

[74] Zhang Y Chen C Choi H Chaisson C Hunter D Niu J Purine-rich foods intake and recurrent gout attacks. *Ann Rheum Dis.* (2012) 71:1448–53. 10.1136/annrheumdis-2011-201215 22648933 PMC3889483

[75] Kanbay M Jensen T Solak Y Le M Roncal-Jimenez C Rivard C Uric acid in metabolic syndrome: From an innocent bystander to a central player. *Eur J Intern Med.* (2016) 29:3–8. 10.1016/j.ejim.2015.11.026 26703429 PMC4826346

[76] Bronikowski AM Meisel RP Biga PR Walters JR Mank JE Larschan E Sex-specific aging in animals: Perspective and future directions. *Aging Cell.* (2022) 21:e13542. 10.1111/acel.13542 35072344 PMC8844111

[77] Sampathkumar NK Bravo JI Chen Y Danthi PS Donahue EK Lai RW Widespread sex dimorphism in aging and age-related diseases. *Hum Genet.* (2020) 139:333–56. 10.1007/s00439-019-02082-w 31677133 PMC7031050

[78] Lu RJ Wang EK Benayoun BA. Functional genomics of inflamm-aging and immunosenescence. *Brief Funct Genomics.* (2022) 21:43–55. 10.1093/bfgp/elab009 33690792 PMC8789293

