# Supplementary material for: Effect of dietary protein content shift on aging in elderly rats by comprehensive quantitative score and metabolomics analysis
Source: Front Nutr. 2022 Nov 3;9:1051964. doi: 10.3389/fnut.2022.1051964 (PMC9673908; doi:10.3389/fnut.2022.1051964)
Supplement: Supplementary file 1 [file Table_1.DOCX]

Supplementary Material

# Supplementary Figures and Tables

## Supplementary Figures


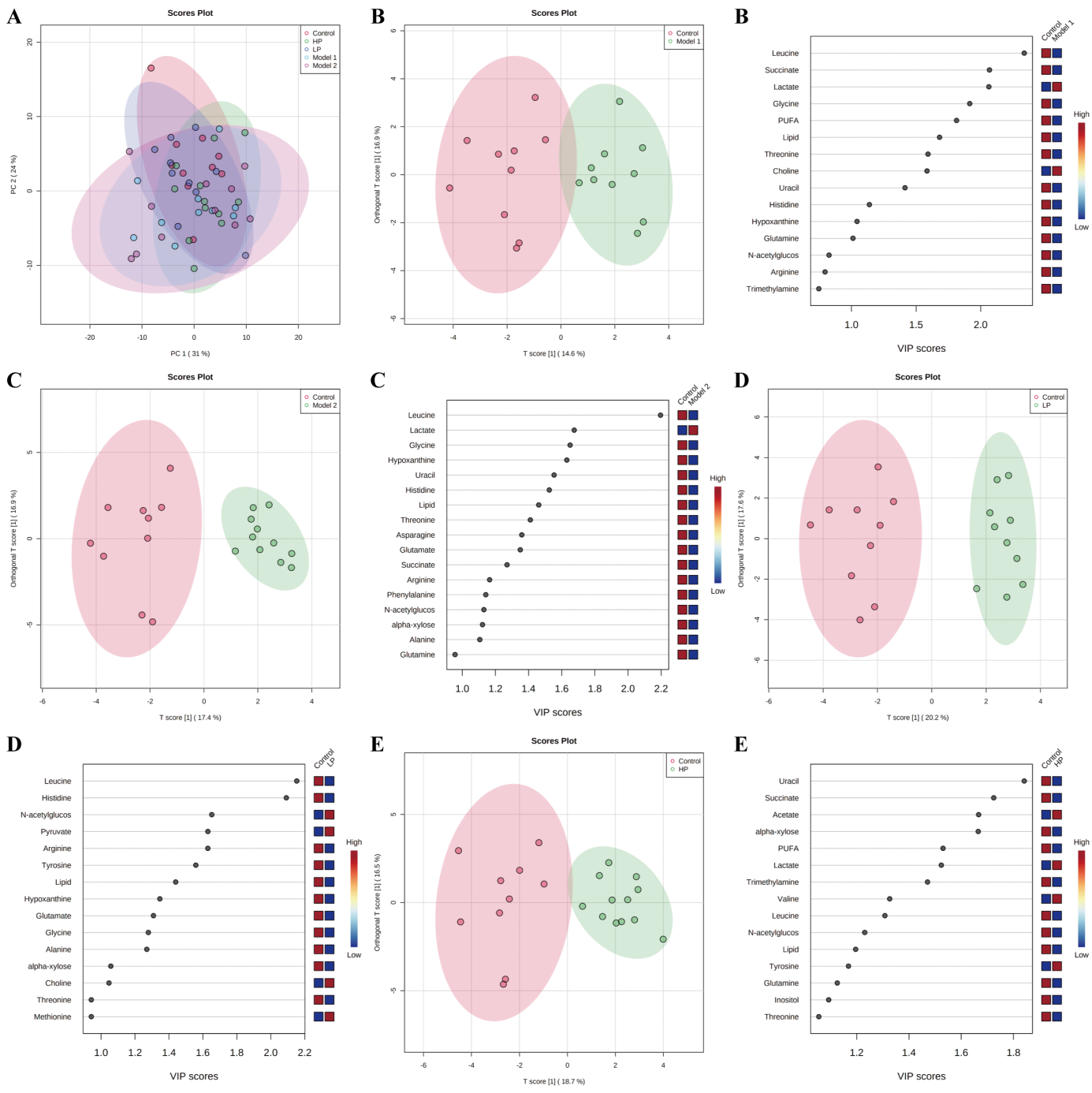


**Supplementary Figure 1.**Fecal metabolomics analysis of rats. (A) Score plots of principal component analysis; (B) OPLS-DA score plots and VIP score rankings between Model1 and control groups; (C) OPLS-DA score plots and VIP score rankings between Model2 and control groups; (D) OPLS-DA score plots and VIP score ranking between low protein diet group and control group ; (E) OPLS-DA score plots and VIP score ranking between high protein diet group and control group. Model parameters: (B), R^2^Y = 0.829, Q^2^ = 0.455, p(CV) = 0.0075; (C), R^2^Y = 0.9, Q^2^ = 0.668, p(CV) < 5 × 10-4; (D), R^2^Y = 0.933, Q^2^ = 0.776, p(CV) < 5 × 10-4; (E), R^2^Y = 0.859, Q^2^ = 0.61, p(CV) = 5 × 10-4. R^2^Y represents the percentage of Y variables being modeled, Q^2^ represents the overall cross-validation value, and p(CV) represents the CV-ANOVA p-value.


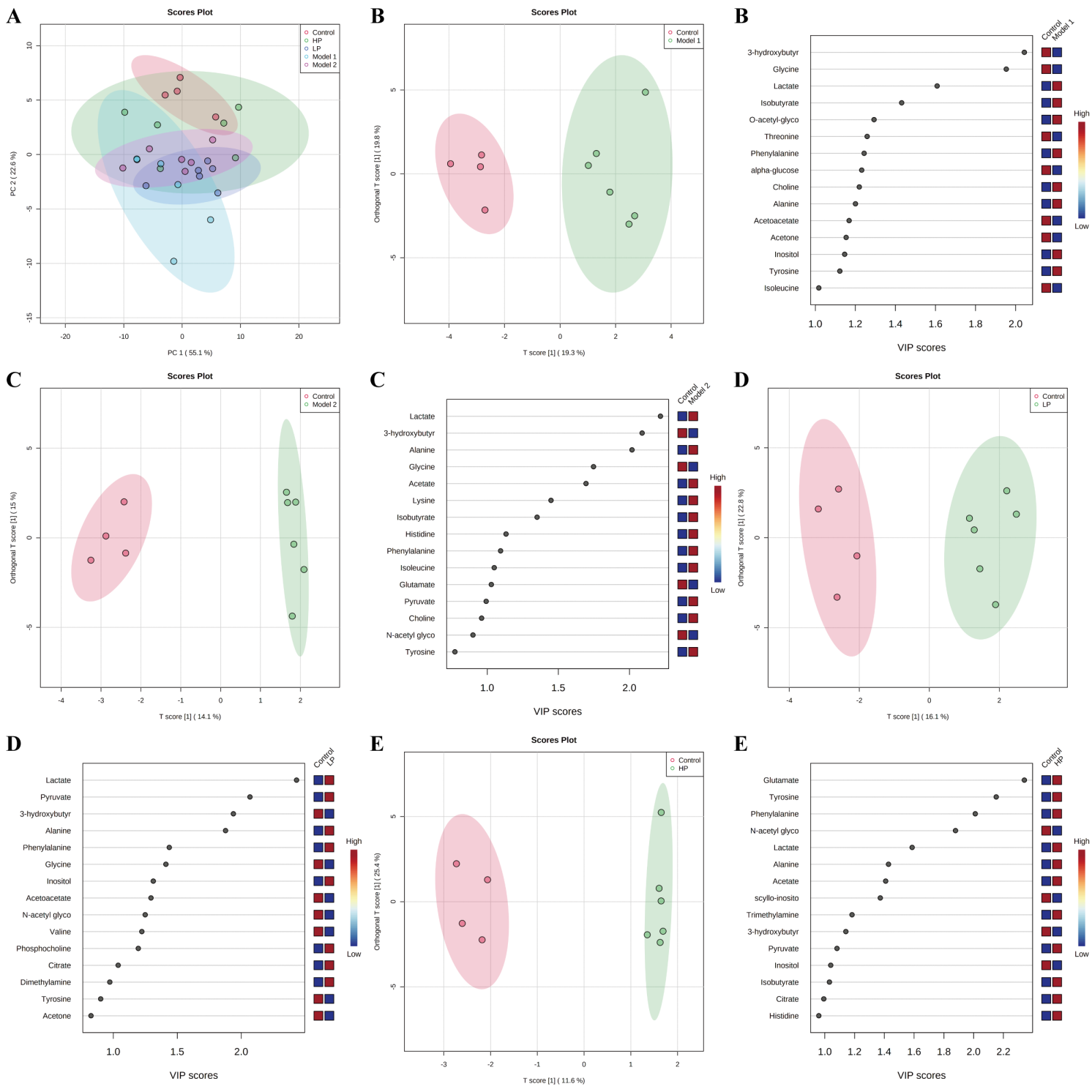


**Supplementary Figure 2.**Serum metabolomics analysis of rats. (A) Score plots of principal component analysis; (B) OPLS-DA score plots and VIP score rankings between Model1 and control groups; (C) OPLS-DA score plots and VIP score rankings between Model2 and control groups; (D) OPLS-DA score plots and VIP score ranking between low protein diet group and control group ; (E) OPLS-DA score plots and VIP score ranking between high protein diet group and control group. Model parameters: (B), R^2^Y = 0.955, Q^2^ = 0.582, p(CV)=0.008; (C), R^2^Y = 0.997, Q^2^ = 0.426, p(CV)=0.042; (D), R^2^Y = 0.933, Q^2^ = 0.776, p(CV)=0.018; (E), R^2^Y = 0.859, Q^2^ = 0.61, p(CV)=0.025. R^2^Y represents the percentage of Y variables being modeled, Q^2^ represents the overall cross-validation value, and p(CV) represents the CV-ANOVA p-value.

## Supplementary Tables

**Table S1.** ^1^H-NMR assignments of major metabolites from fecal samples of rats.

| No. | Metabolites | δ^1^H/(multiplicity) | No. | Metabolites | δ^1^H/(multiplicity) |
| --- | --- | --- | --- | --- | --- |
| 1 | Lipid | 0.89 (t) | 23 | Trimethylamine | 2.86 (s) |
| 2 | Butyrate | 0.91 (m) | 24 | Asparagine | 3.03 (m) |
| 3 | Isoleucine | 0.93 (t), 1.01 (d) | 25 | Histidine | 3.14 (m), 7.88 (s) |
| 4 | Leucine | 0.96 (d), 0.98 (d) | 26 | Choline | 3.20 (s) |
| 5 | Valine | 1.04 (d) | 27 | β-xylose | 3.23 (s) |
| 6 | Propionate | 1.06 (t), 2.19 (q) | 28 | Taurine | 3.25 (t) |
| 7 | Ethanol | 1.17 (t), 3.64 (q) | 29 | Betaine | 3.27 (s), 3.89 (s) |
| 8 | Lactate | 1.33 (d), 4.12 (q) | 30 | Phenylalanine | 3.30 (m), 3.97 (d), 4.06 (dd), 7.33 (d), 7.42 (m) |
| 9 | Alanine | 1.48 (d), 3.78 (m) | 31 | Methanol | 3.37 (s) |
| 10 | Citrulline | 1.56 (m) | 32 | α-glucose | 3.43 (dd), 3.84 (dd), 5.24 (d) |
| 11 | Arginine | 1.72 (m), 3.76 (m) | 33 | β-glucose | 3.47 (dd), 3.75 (m) |
| 12 | Acetate | 1.92 (s) | 34 | Inositol | 3.53 (dd) |
| 13 | Proline | 2.05 (m), 3.34 (m) | 35 | Glycine | 3.56 (s) |
| 14 | N-acetylglucosamine | 2.06 (s) | 36 | Threonine | 3.58 (d), 4.22 (m) |
| 15 | Glutamate | 2.08 (m), 2.36 (m) | 37 | Glycolate | 3.94 (s) |
| 16 | Methionine | 2.14 (s) | 38 | α-xylose | 5.2 (t) |
| 17 | Glutamine | 2.15 (m), 2.47 (m), 3.79 (t) | 39 | Uracil | 5.81 (d), 7.54 (d) |
| 18 | Pyruvate | 2.37 (s) | 40 | Fumarate | 6.52 (s) |
| 19 | Succinate | 2.41 (s) | 41 | 3-hydroxyphenyl propionic acid | 6.8 (m) |
| 20 | PUFA | 2.65 (s) | 42 | Tyrosine | 6.92 (d), 7.2 (d) |
| 21 | Aspartate | 2.69 (dd), 2.83 (dd), 3.91 (dd) | 43 | Hypoxanthine | 8.19 (s), 8.21 (s) |
| 22 | Dimethylamine | 2.73 (s) |  |  |  |

Note: s, singlet; d, doublet; t, triplet; q, quartet; dd, double doublet; m, multiplet.

**Table S2.** ^1^H-NMR assignments of major metabolites from serum samples of rats.

| No. | Metabolites | δ^1^H/(multiplicity) | No. | Metabolites | δ^1^H/(multiplicity) |
| --- | --- | --- | --- | --- | --- |
| 1 | Lipid | 0.87 (m), 1.28 (m) | 21 | Dimethylamine | 2.75 (s) |
| 2 | Isoleucine | 0.94 (d) | 22 | Trimethylamine | 2.91 (s) |
| 3 | Leucine | 0.96 (d), 0.98 (d) | 23 | Creatine | 3.03 (s), 3.94 (s) |
| 4 | Valine | 1.04 (d) | 24 | Choline | 3.20 (s) |
| 5 | Isobutyrate | 1.07 (d) | 25 | Phosphorylcholine | 3.21 (s) |
| 6 | 3-hydroxybutyrate | 1.18 (d), 2.31 (d), 2.40 (d) | 26 | β-glucose | 3.23 (dd), 3.46 (m), 3.77 (dd), 3.91 (dd) |
| 7 | Lactate | 1.32 (d), 4.11 (q) | 27 | Trimethylamine oxide | 3.25 (s) |
| 8 | Alanine | 1.47 (d) | 28 | Betaine | 3.26 (s) |
| 9 | Lysine | 1.71 (m), 1.87 (m), 3.00 (s) | 29 | Scyllo-inositol | 3.35 (s) |
| 10 | Acetate | 1.91 (s) | 30 | α-glucose | 3.41 (m), 3.54 (dd), 3.71 (m), 3.83 (dd), 3.85 (m), 5.23 (d) |
| 11 | N-acetylglucosamine | 2.04 (s) | 31 | Glycine | 3.55 (s) |
| 12 | Glutamate | 2.09 (m), 2.35 (m) | 32 | Threonine | 3.57 (d) |
| 13 | Glutamine | 2.11 (m) | 33 | Propanetriol | 3.62 (s) |
| 14 | O-acetyl glycoprotein | 2.14 (s) | 34 | Inositol | 3.63 (s) |
| 15 | Acetone | 2.23 (s) | 35 | Arginine | 3.79 (m) |
| 16 | Acetoacetate | 2.27 (s) | 36 | 1-methylhistidine | 3.95 (d) |
| 17 | Pyruvate | 2.36 (s) | 37 | Histidine | 3.97 (d), 7.03 (s), 7.73 (s) |
| 18 | Succinate | 2.42 (s) | 38 | Tyrosine | 6.88 (d), 7.18 (d) |
| 19 | Carnitine | 2.44 (dd) | 39 | Phenylalanine | 7.31 (s), 7.41 (s) |
| 20 | Citrate | 2.53 (d), 2.69 (d) | 40 | Formate | 8.45 (s) |

Note: s, singlet; d, doublet; t, triplet; q, quartet; dd, double doublet; m, multiplet.
